# Obituary Notice of Professor Brown

**Published:** 1830

**Authors:** 


29. *Obituary of Professor Brown.* Died at the residence of his brother-in-law, Col. Percy, near Huntsville, Alabama, on the 12th of January last, (1830,) Doctor SAMUEL BROWN, formerly professor of the Theory and Practice of Medicine in Transylvania University. His disease, as we are informed by our friend Dr. Picket, one of his medical attendants, was Apoplexy; to which, from his sanguineo-lymphatic temperament, he had a predisposition. He was upwards of 60 years old.

Dr. Brown was the son of a Presbyterian clergyman, and born in Virginia; where he studied the elements of his profession, but completed his education in the University of Edinburgh, in the latter part of the last century. He commenced the practice of the profession on the banks of the Potomac; but about the year 1796 emigrated to Lexington, Ky.; where he resided for many years, and established a high reputation. From Lexington he emigrated to New-Orleans, where he prosecuted his profession for a time, when he married Miss Percy, of Mississippi, and went to reside in that state. This lady dying, soon aftarwards, left him with two children; when he returned to Kentucky, and at length removed to Alabama. From that state, about the year 1817, he visited Philadelphia, where he continued to reside, a great part of his time, till the autumn of 1828, when he returned to Alabama, and remained there up to the time of his death.

In the month of January, 1819, Dr. Brown was appointed, by the legislature of Ohio, in the act incorporating the Medical College of Ohio, one of its professors, but did not accept. In the succeeding spring, he was offered the chair of the Theory and Practice of Medicine, in Transylvania University, and in the following autumn entered on its duties. This station he held till the end of 1825, when he resigned, having delivered six courses of lectures. The summers of 1824 and 1828 he spent in London and Paris.

While he continued in the practice of his profession, Dr. Brown was greatly esteemed by his patients, for his skill and suavity. He was a gentleman of extensive information, both in and out of the professsion; and possessed great natural capacities for acquiring scientific knowledge. His mind was brilliant and penetrating, but not remarkable for patient and protracted analysis. As a lecturer he was brief and desultory, but abounded in valuable facts, ingenious suggestions, vivid conceptions, and most apposite anecdotes. Hence his pupils were always pleased as well as instucted, and allowed themselves to be reconciled to his deficiencies of method, by the value of his insulated facts and speculations.

Professor Brown was the founder of the association, denominated the *Kappa Lambda Society of Hippocrates*; the number of whose members, especially, in the western and southern portions of the United States, is considerable. Its object is to unite the best members of the profession, on every feasible and honorable plan for elevating and sustaining its dignity. It does not assume to itself more learning; but learning, means and objects, of a kind more consonant with professional excellence and honor; and hence its motto—

'*Non doctior, sed meliore imbutus doctrina*'

which has since been adopted into the title page of a Journal, established under a resolution and conducted on the principles of the society, by an able and honorable committee of its members.

Like many other experienced and learned physicians, Dr. Brown wrote but little. His longest production of any kind, is a paper on the salt petre caves of Kentucky, published in one of the early volumes of the Transactions of the American Philosophical Society. On subjects strictly professional, he contributed two or three short papers, to the Medical Repository, edited by Dr. Mitchill, which afforded good evidence of practical tact, and are calculated to exeite a regret, that he should not have left behind him a more copious record of his ample experience.

In manners and social qualities, the deceased was polished and facinating. His personal acquaintance with the students of the University at Lexington, was extensive and made him pre-eminently, a favourite with all the classes. Widely dispersed as they now are, he will long be recollected with thusiasm, throughout the states of the West and South, while society will enjoy the benefits of the sound practical knowledge which he imparted, and the glowing philanthropy which he inspired.

